# The complete mitochondrial genome of *Neritina violacea*

**DOI:** 10.1080/23802359.2019.1662744

**Published:** 2019-09-11

**Authors:** Pengliang Wang, Peng Zhu, Haiping Wu, Youhou Xu, Yongyan Liao, Hong Zhang

**Affiliations:** Guangxi Key Laboratory of Beibu Gulf Marine Biodiversity Conservation, Beibu Gulf University, Qinzhou, China

**Keywords:** *Neritina violacea*, Mitochondrial genome, Illumina sequencing

## Abstract

*Neritina violacea* is a common and important component of mangrove ecosystem. In this study, the mitogenome of *N. violacea* was determined for the first time using next-generation sequencing; the overall base components of mitogenome consisting of 15,710 bp was 31.37% for A, 34.91% for T, 19.47% for G, 14.25% for C, and its GC content was 33.72%. The mitogenome was composed of 13 protein-coding genes, 22 tranfer RNAs, and 2 ribosomal RNAs. Polygenetic analysis showed that the *N. violacea* was more close to *N. Usnea* and *Theodoxus fluviatilis*. We speculated that the *N. violacea* was evolved from freshwater species.

The *Neritina violacea* is one member of the family *Neritidae*, dominating in mangrove forests in the coast of south china sea, in marine and brackish conditions (Uribe et al. 2016). Mitochondria play a vital role in energy metabolism and contain its own genome. Due to the nature of maternal inheritance, non-recombination and high substitution rate (Ingman et al. 2000), the mitogenome is a very useful tool for the study of population genetics and molecular phylogenetics studies. Therefore, the complete mitogenome of *N. violacea* was reported here, which could provide important genomic information for analyzing the evolution relationship of family *Neritidae*, though the molecular phylogeny of some species of the *Neritideae* based on two mitochondrial genes (COI and 16S rRNA) (Quintero-galvis and Castro 2013).

The individual of *N. violacea* was collected from XiNiuJiao region, Qinzhou, Guangxi, China (21.828 N, 108.601E) and its genomic DNA was extracted, then stored at −20 °C refrigerator in the herbarium of ocean college in Beibu Gulf University (N.V.002). Paired-end library (450 bp) was constructed and sequenced using Illumina Hiseq4000. 19,506 raw reads and 2,925,900 total bases were obtained, with average reads depth of 186.2X. After low-quality reads were removed from the raw reads, the clean reads were generated and assembled into mitochondrial genome using the chloroplast and mitochondrion assemble (CMA)V1.1.1 software. Protein-coding genes and rRNA genes were annotated with blast+ (2.5.0) with allied species and tRNAs were predicted with tRNAscan-SE v2.0 (Lowe and Chan 2016). Our findings revealed that the circular genome was 15,710 bp in size and comprises 13 protein-coding genes (PCGs), 2 rRNAs genes and 22 tRNAs genes. Of these genes, 7 PCGs and 9 tRNAs were located on heavy strand, the others on the light strand. The results showed that the gene composition and arrangement are more close to *N. Usnea* and *Theodoxus fluviatilis*. The contents of A, T, G, and C in mitochondrial genome of this species were 31.37, 34.91, 19.47, and 14.25%, respectively. An overall GC content of whole mitochondrial genome is 33.72%. The sequence data were available in GenBank (KY021066). The phylogenetic analysis was conducted among 20 mitogenomes for *N. Violacea* and an entire mitogenome of *Owenia fusiformis* as an outgroup using maximum likelihood (ML) method. The best-fit model of evolution for the coding genes was selected by jmodeltest2 (Darriba et al. 2012). RAxML v8.0.0 (Stamatakis 2014) was employed to construct the phylogenetic tree with 1000 bootstrap (Figure 1). Phylogenetic analyses indicated the presence of two distinct clades in Neritidae, and the *N. violacea* was more closed to *N. Usnea* and *Theodoxus fluviatilis* who can live in both freshwater and brackish water (Kangas and Skoog 1978). Furthermore, *N. violacea* has genetic relationship with *Potamopyrgus antipodarum*, a kind of very small or minute freshwater snail. We speculated that the *N. violacea* was evolved from freshwater species. The compete mitogenome of *N. violacea* provided important genetic information for understanding phylogenetic relationships of *Neritidae* and figured out how the *N. violacea* co-evolved in the mangrove ecosystem.

**Figure 1.**
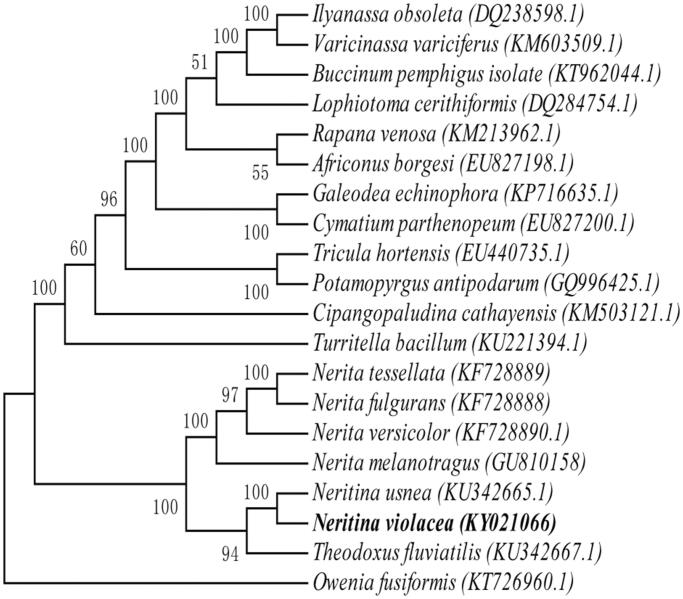
Phylogenetic relationships of *Neritina violacea* with another 19 mitochondrial genomes using NJ method. GenBank accession number: *Ilyanassa obsolete (DQ238598.1), Varicinassa variciferus (KM603509.1), Buccinum pemphigus isolate (KT962044.1), Lophiotoma cerithiformis (DQ284754.1), Rapana venosa (KM213962.1), Africonus borgesi (EU827198.1), Galeodea echinophora (KP716635.1), Cymatium parthenopeum (EU827200.1), Tricula hortensis (EU440735.1), Potamopyrgus antipodarum (GQ996425.1), Cipangopaludina cathayensis (KM503121.1), Turritella bacillum (KU221394.1), Nerita tessellate (KF728889), Nerita fulgurans (KF728888), Nerita versicolor (KF728890.1), Nerita melanotragus (GU810158), Neritina usnea (KU342665.1), Theodoxus fluviatilis (KU342667.1), Owenia fusiformis (KT726960.1).*
